# Molecular Identification of *Fasciola* Isolated from the Liver of Meat Animals in Fars Province, Iran

**DOI:** 10.1155/2022/4291230

**Published:** 2022-03-26

**Authors:** Aminallah Saadatnia, Kavous Solhjoo, Mohamad Hasan Davami, Saber Raeghi, Ahmad Abolghazi

**Affiliations:** ^1^Department of Medical Parasitology and Mycology, Zoonoses Research Center, Jahrom University of Medical Sciences, Jahrom, Iran; ^2^Department of Medical Parasitology, Yasuj University of Medical Sciences, Yasuj, Iran; ^3^Department of Laboratory Sciences, Maragheh University of Medical Sciences, Maragheh, Iran; ^4^Department of Medical Parasitology, Hamedan University of Medical Sciences, Hamedan, Iran

## Abstract

**Background:**

*Fasciola hepatica* and *Fasciola gigantica* are flatworms that infect animals and humans. *Fasciola* is the parasite of the liver or bile ducts and intestines of mammals, where such animals are known as their “definite hosts.” The study aims to detect the genotype of *Fasciola* spp. from the livers of meat animals by using RFLP-PCR in samples collected from Fars province.

**Methods:**

Sixty *Fasciola* spp. samples were collected from infected slaughtered animals in three counties of Fars province, Iran (Jahrom, Nourabad Mamasani, and Kazeroun).Genomic DNA was extracted by the conventional phenol-chloroform method. For the study, PCR-RFLP and sequence analysis of the first nuclear ribosomal internal transcribed spacer (ITS1) region from *Fasciola* species were used to conduct the study.

**Results:**

The fragment of about 700 bp in all the *Fasciola* samples was amplified. In total, 43 samples of *Fasciola gigantica* and 17 samples of *Fasciola hepatica* were identified.

**Conclusion:**

The dominant *Fasciola* species in this region is *Fasciola gigantica*. Hence, it seems that hygienic policies should be developed to prevent and control fascioliasis because of the dominant species, *Fasciola gigantica*.

## 1. Introduction

*Fasciola hepatica* and *Fasciola gigantica* are flatworms and belong to the trematode class and Digenea subclass, which cause infection in animals and humans [1]. *Fasciola* spp. is the parasite of the liver or bile ducts and intestines of mammals; such animals are known as their “definitive hosts” [2]. The geographical distribution of Fascioliasis is worldwide, especially where the breeding of sheep, cows, and goats is common [3, 4]. Fascioliasis is the most common helminth infection in tropical countries, with a 90% prevalence. Human contamination with this parasite affects more than 2 million people worldwide, distributed in more than 60 countries. About 180 million people are at high risk of being exposed to this disease around the world [4, 5]. This parasite causes a considerable economic loss throughout the globe; it approaches two billion dollars per year and is one of the most important problems in human and animal health [6]. Several cases have been reported of the human disease caused by this parasite in some countries, including Iran. Iran has always involved the problems created by this parasite for framers, villagers, and industrial producers of animals [7–9]. In some countries, the distribution of Fascioliasis overlaps, and it has been reported in livestock or humans in Asian countries such as Iran. Human fascioliasis in Iran was sporadic up to 1987. But in 1988 and 1989, two large epidemics occurred in northern Iran [10, 11]. Fars province (Jahrom, Nourabad Mamasani, and Kazeroun) is one of the best hotspots for fascioliasis in Iran [12]. Two species of *Fasciola hepatica* and *Fasciola gigantica* have their own special life cycles in different regions of Iran, and to complete the parasite's life cycle, the presence of the main species of vector snails as the intermediate hosts is required [6]. Traditional policies for fasciolosis prevention and control in each area necessitate an accurate distinction of dominant species. Molecular identification of *Fasciola* spp. is useful as it can help disease monitoring, diagnosis, and control of the parasite [13]. A common diagnosis of these two species is based on the morphological features of adult worms and eggs. While the distinction between the two species of *Fasciola* spp. is not valid based on the clinical, pathological symptoms, immunological, and fecal methods [14], these conventional methods were replaced by new molecular techniques. The molecular techniques have high efficiency and sensitivity compared to the routine methods because the worm genome is examined. In some Iranian regions, PCR-RFLP and PCR methods using the genetic areas of ITS2, ITS1, and ribosomal DNA for the determination of the genotype and the phylogenetic analysis of *Fasciola* spp. were used, and it was found that both species of *Fasciola* are present in Iran [15]. As the characterization of the *Fasciola* spp. is very important for the control of fascioliasis in humans and animals [3], this study was carried out to identify and differentiate *Fasciola* spp. isolated from the livers of meat animals by PCR-RFLP of ITS1 analysis in Fars province.

## 2. Methods

**Figure 1:**
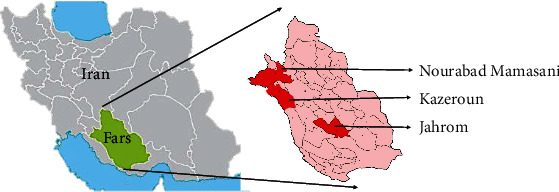
Geographic region image of Fars province (Jahrom, Nourabad Mamasani, and Kazeroun) in the south of Iran.

This descriptive cross-sectional study was carried out on 60 *Fasciola* spp. adult flukes collected from the livers of livestock (cow, sheep, and goat) in three counties of Fars Province in Iran (Jahrom, Nourabad Mamasani, and Kazeroun) (Figure 1). The infected livers were collected and transported to the laboratory of parasitology, where the liver was dissected with a scalpel (Bistouri) and the flukes (28, 10, and 22 samples) were collected from the livers of cows, goats, and sheep, respectively). The flukes were identified as *Fasciola* spp. morphologically, thoroughly washed with phosphate-buffered saline (PBS), and kept in 70% ethanol at room temperature until the extraction of genomic DNA.

### 2.1. DNA Extraction

To extract the DNA, about 10 gr of parasite tissue was taken from the margin with a scalpel (Bistouri). Then, the alcohol was allowed to exhaust for several minutes before being put into the 1.5 microtube. Genomic DNA was extracted from the flukes using the phenol-chloroform method. The extracted DNA was kept at –20°C until it was used in the PCR. Polymerase chain reaction (PCR) using the lyophilized PCR PreMix manufactured by BIONEER Company, South Korea, was carried out in 20 *μ*l of the total volume, containing 10 *μ*l of PreMix, 4 *μ*l of distilled water, 4 *μ*l of DNA, 1 *μ*l of forward primer, and 1 *μ*l of reverse primer. Then, the Its1 gene amplification was made using the specific primers ITS1-F primer and primer sequence of 5-TTGCGCTGATTACGTCCCTG-3 and ITS1-R primer with a primer sequence of 5-TTGGCTGCGCTCTTCATCGAC-3 [16] with a temperature plan of (94°C for 3 min), annealing [(94°C for 90 sec, 55°C for 90 sec, and 72°C for 2 min and 30 cycles], and extension step: (72°C for 10 min), and the Its1 gene was replicated in 700 bp in length [17]. To confirm the PCR implementation in each stage, the obtained products were electrophoresed on a 1.5% agarose gel and compared to the marker. The size of other products was measurable. A RFLP-PCR was conducted for the identification of parasite species. Based on RFLP, 5 *μ*l of *Fasciola* ITS1 PCR product, 2.5 *μ*l of supplied restriction enzyme buffer, 5 *μ*l of Rsa1 restriction enzyme diluted, and distilled water up to 25 *μ*l were implemented. According to the manufacturer, instruction, the tubes were incubated at 37°C for 7 h, to ensure full cutting of fragments. For analyzing the digestion products, 15 *μ*l of each product in addition to 2 *μ*l of loading buffer was electrophoresed on 3% agarose gel [17].

## 3. Results

**Figure 2:**
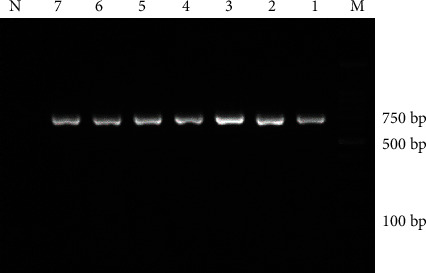
PCR results using the ITS forward and reverse primers for ITS1 gene fragment obtained from the *Fasciola* samples in this study, line marker M, lines 1-7 of *Fasciola* samples and line N negative control.

**Figure 3:**
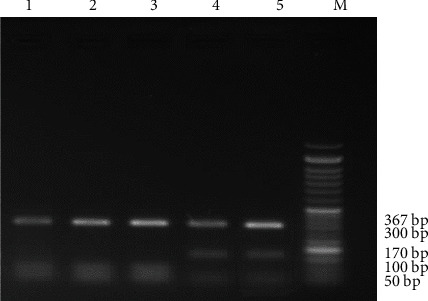
RFLP pattern from PCR products for *Fasciola hepatica* and *Fasciola gigantica* with digestive enzyme Rsa1. M marker 50 bp, lines 4 and 5 of *Fasciola gigantica*, lines 2, 1, and 3 of *Fasciola hepatica.*

Genomic DNA was extracted from 60 samples, which could amplify an ITS1 gene fragment of about 700 bp in all the isolates. Then, the Rsa1 enzyme is used to determine the *Fasciola* species. I was able to distinguish between the two types of *Fasciola* based on patterns of fragments digested with Rsa1. The RFLP patterns from *Fasciola gigantica*, which had 4 cutting sites, were predicted to be separated into fragments of 367, 172, 59, 54, and 28 bp in the amplicons, while 5 fragments were produced by 5 cutting sites from *F. hepatica* including 367, 104, 68, 59, 54, and 28 bp in the amplicons. Based on the PCR-RFLP, all samples were classified as parasite species. In total, out of 60 samples, 43 (71.6%) and 17 (28.4%) samples were *Fasciola gigantica* and *Fasciola hepatica*, respectively (Figures 2 and 3). Of the 43 positive *Fasciola gigantica* samples, 17 were sheep, 19 were cow, and 7 were goat and of the 17 positive *Fasciola hepatica* samples were 5 sheep, 9 cows, and 3 goats.

## 4. Discussion

Fascioliasis caused by *Fasciola* species (*Fasciola hepatica* and *Fasciola gigantica*) is one of the most important zoonotic diseases in the world [7]. Fascioliasis is a very important veterinary problem because it causes great economic losses in the livestock industry, especially in cattle and sheep, and due to the parasite species and host type, it can be very deadly in sheep and an asymptomatic infection in cows [18]. In Iran, finding a suitable way to prevent and control fascioliasis has been a problem in hygiene [8]. The effective fight against this disease has resulted in the precise identification of the parasite and its dominant species. In other studies, in different areas of the country, to specify the dominant species of *Fasciola*, different genes, including Its1, Its2, Nod, and Cox1, are used to diagnose *Fasciola*. For accurate diagnosis of the *Fasciola* species, various enzymes such as Rsa1 and Kpn1 are used. Rokni et al. [19], Aryaeipour et al. [20], and [21] used the gene Its1 to identify the *Fasciola* [22][19–21]). Like in the aforementioned studies, in this research, the *Fasciola* parasites were diagnosed by Its1 [16]. In the present study, the Rsa1 enzyme was used for certain determinations of *Fasciola* species. In studies by [20] and (Mirahmadi et al. 2018), they investigated the *Fasciola* taken from the domestic animals in Ardabil and Sistan-and-Balouchestan Provinces, respectively, by Its1 and enzyme Rsa1. They reported that the number of both species was the same, while in this study, the number of diagnosed *Fasciola gigantica* was greater than the *Fasciola hepatica* [23, 24].

(Piri et al. 2018) studied the *Fasciola* in Hamedan, and by Its1 and enzyme Rsa1, they reported that out of all samples, the dominant *Fasciola* species in this region was *Fasciola hepatica*. In this study, the number of diagnosed *Fasciola gigantica* was more than the *Fasciola hepatica*, which was not similar to this study [25]. (Mir et al. 2019) investigated the *Fasciola* in Zabol County, and by Its1 and enzyme Rsa1, they stated that out of 70 samples, 63 and only 7 samples had been *Fasciola* hepatica and diagnosed *Fasciola gigantica*, respectively, and the dominant *Fasciola* species in this region was the *Fasciola gigantica* [26]. In this study, the number of *Fasciola gigantica* was also greater than that of *Fasciola hepatica*, indicating the dominance of *Fasciola gigantica*, the same as in the study in Zabol. Due to endemic fascioliasis in Iran and the existence of suitable conditions for the transmission of this disease, fascioliasis has become an important part of the hygienic priorities of Iran. [27]. The economic losses imposed by fascioliasis are among the factors which require the development of appropriate policies for preventing and controlling this disease in each area [7]. The efficient struggle against this disease requires the exact identification of this parasite and its dominant species. This has led to various studies in different parts of the country to specify the dominant species of *Fasciola*. Like other studies in Iran, this research was also carried out using gene Its1 and the enzyme Rsa1 to perform RFLP-PCR [7, 8]. The results of this study and others indicate the diagnostic power of the PCR-RFLP method and the appropriateness of gene Its1 and enzyme Rsa1 in diagnosing the *Fasciola* species (Kobra et al., 2018). The results of this and other studies in Iran have shown that both types of *Fasciola hepatica* and *Fasciola gigantica* are present in Iran and depending on the type of final host, the type of specific intermediate host snails, weather conditions, and local plants contaminated by *Fasciola* and dominant species, the cause of disease is different in each region [8, 28].

## 5. Conclusion

The dominant *Fasciola* species in this region is *Fasciola gigantica*. Hence, it seems that hygienic policies should be developed to prevent and control Fascioliasis because of the dominant species, i.e., *Fasciola gigantica*.

## Data Availability

No data were used to support this study.
